# Salivary Biomarkers for Diagnosis of Inflammatory Bowel Diseases: A Systematic Review

**DOI:** 10.3390/ijms21207477

**Published:** 2020-10-10

**Authors:** Kacper Nijakowski, Anna Surdacka

**Affiliations:** Department of Conservative Dentistry and Endodontics, Poznan University of Medical Sciences, 60-812 Poznan, Poland; annasurd@ump.edu.pl

**Keywords:** inflammatory bowel disease, saliva, biomarkers

## Abstract

Saliva as a biological fluid has a remarkable potential in the non-invasive diagnostics of several systemic disorders. Inflammatory bowel diseases are chronic inflammatory disorders of the gastrointestinal tract. This systematic review was designed to answer the question “Are salivary biomarkers reliable for the diagnosis of inflammatory bowel diseases?”. Following the inclusion and exclusion criteria, eleven studies were included (according to PRISMA statement guidelines). Due to their heterogeneity, the potential salivary markers for IBD were divided into four groups: oxidative status markers, inflammatory cytokines, microRNAs and other biomarkers. Active CD patients manifest decreased activity of antioxidants (e.g., glutathione, catalase) and increased lipid peroxidation. Therefore, malondialdehyde seems to be a good diagnostic marker of CD. Moreover, elevated concentrations of proinflammatory cytokines (such as interleukin 1β, interleukin 6 or tumour necrosis factor α) are associated with the activity of IBD. Additionaly, selected miRNAs are altered in saliva (overexpressed miR-101 in CD; overexpressed miR-21, miR-31, miR-142-3p and underexpressed miR-142-5p in UC). Among other salivary biomarkers, exosomal PSMA7, α-amylase and calprotectin are detected. In conclusion, saliva contains several biomarkers which can be used credibly for the early diagnosis and regular monitoring of IBD. However, further investigations are necessary to validate these findings, as well as to identify new reliable salivary biomarkers.

## 1. Introduction

Saliva is an opalescent biological fluid containing a mixture of secretions from three pairs of major (including parotid, submandibular and sublingual) and many minor salivary glands, as well as gingival crevicular fluid. It performs numerous functions: protective, digestive, defensive and diagnostic. Saliva, like blood, contains numerous enzymes, hormones or antibodies [1]. Thanks to the new techniques, such as molecular diagnostics or nanotechnology, the small volume of fluid and low concentrations of examined elements are no longer an obstacle [2]. Saliva is collected easily and non-invasively, eliminating the stress caused to patients during blood sampling. Saliva collection is safe even for less qualified medical personnel and does not require—like clotting blood—special conditions before delivery to the laboratory [3]. New future molecular technologies focus on the analysis of proteins and nucleic acids contained in saliva [4,5,6]. Promising future solutions are lab-on-a-chip (LOC) and point-of-care (POC) technologies, which would be portable, miniaturised devices giving immediate results, based on the principles of conventional ELISA tests or nucleic acid hybridisation [7]. Saliva has an impressive potential in the diagnostics of several systemic disorders, including oncological, cardiovascular, endocrine, autoimmune, neurological, infectious or just gastrointestinal diseases [8].

Moreover, it should be emphasized that oral inflammation may change the composition of saliva and interfere with the expression of selected diagnostic proteins. The common oral diseases include dental caries and periodontal diseases. Vitorino et al. [9] identified higher concentrations of amylase, immunoglobulin A, lactoferrin, lipocalin, cystatins and proline rich acid proteins (PRPs) in caries-free patients. Rudney et al. [10] indicated that elevated levels of staterin and truncated cystatin S may be associated with an increased risk of caries development. Furthermore, Fábián et al. [11] reviewed salivary proteins linked with periodontitis. These include immunoglobulins, heat shock protein Hsp70, cystatin S, amylase, calprotectin, histatin, lysozyme, lactoferrin, defensin, peroxidase, PRPs and mucins. Both active and passive smoking influence the condition of periodontium. In active smokers, Kibayashi et al. [12] observed significantly decreased concentrations of prostaglandin E2, lactoferrin, albumin, aspartame aminotransferase, lactose dehydrogenase and alkaline phosphatase. In passive smokers, Nishida et al. [13] determined reduced levels of interleukin 1β, albumin and aspartame aminotransferase.

Inflammatory bowel diseases (IBD) comprise chronic inflammatory disorders of the gastrointestinal tract, affecting millions worldwide [14]. Despite many investigations, the exact etiopathogenesis remains unknown. Potential factors involve genetic predisposition, environmental conditions and immunological dysfunctions. The main forms of IBD are Crohn’s disease (CD) and ulcerative colitis (UC). Although transmural inflammation in CD may affect any part of the gastrointestinal tract from the oral cavity to the rectum, it occurs most frequently in the terminal ileum or the large intestine. In contrast, UC usually concerns only the large intestine and is limited to the mucosal layer [15]. The active IBD may manifest with symptoms, such as chronic diarrhoea, abdominal pain, weight loss, fever or even severe complications, e.g., intestinal fistulas or intra-abdominal abscess [16]. The diagnosis is confirmed based on the endoscopy and histological examination of the inflamed mucous membrane biopsy (current gold standard). In IBD patients, it may occur oral lesions—specific (e.g., for CD cobblestoning, mucosal tags or deep linear ulcerations, and for UC pyostomatitis vegetans) or nonspecific, such as aphthous stomatitis, angular cheilitis or atrophic glossitis [17].

The present systematic review was designed in order to answer the question “Are salivary biomarkers reliable for the diagnosis of inflammatory bowel diseases?”, formulated according to the PICO (“population”, “intervention”, “comparison”, “outcome”).

## 2. Results

**Figure 1:**
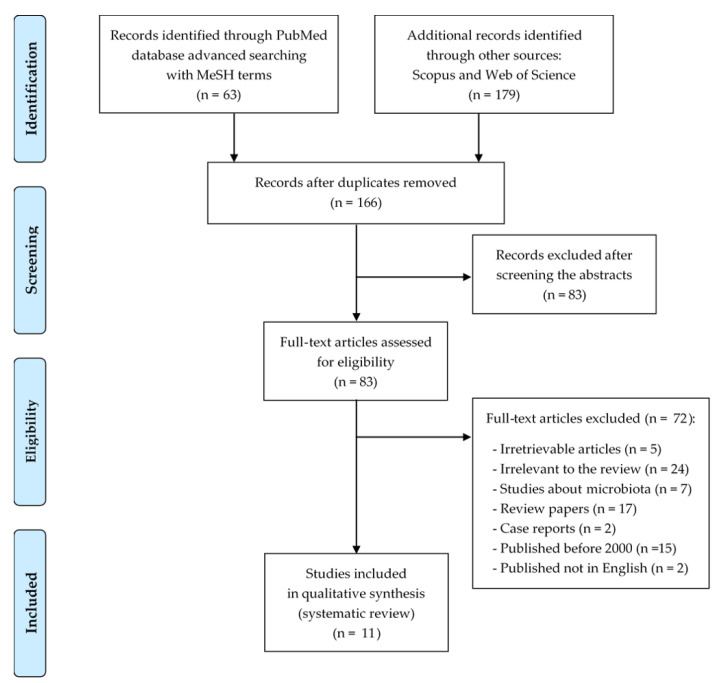
PRISMA flow diagram presenting the detailed search strategy.

In this systematic review, eleven studies following the search criteria were included—data were collected in seven different countries, from a total of 631 participants (including 255 patients with Crohn’s disease and 158 with ulcerative colitis). Figure 1 shows the detailed selection strategy of the articles. The inclusion and exclusion criteria are presented in the section Materials and Methods. 

From each eligible study included in the present review, data about its general characteristics such year of publication and setting, involved participants, pharmacological treatment prior to the study and smoking habits, potential salivary biomarkers for IBD were collected—Table 1. The majority of studies had information about the exclusion of patients with periodontal disease as the primary exponent of inflammation in the oral cavity. Table 2 presents the types of saliva, methods of collection, centrifugation, storing and laboratory analysis. All of the studies took into consideration whole saliva specimens. Only five studies described the precise instructions given to patients before saliva collection. Saliva centrifugation methods were rather heterogeneous but the most frequent method of sample storing was freezing at −80 °C. Additionally, the parameters of statistical significance for selected salivary biomarkers were reported in Table 3. Only two studies performed ROC analysis of the potential biomarkers for IBD.

## 3. Discussion

In the following discussion, the potential salivary markers for IBD were divided into four subgroups: oxidative status markers, inflammatory cytokines, microRNAs and other biomarkers. This section structure was established intentionally because of the considerable heterogeneity of discussed studies.

### 3.1. Oxidative Status Markers

Oxidative stress reflects the imbalance between excessively produced reactive oxygen species (ROS) and the insufficient activity of antioxidants which may lead to cell and tissue damage. Among the most important salivary antioxidants, the following should be mentioned—uric acid, albumin and transferrin. They play a key role by scavenging free oxygen radicals (e.g., peroxyl or hydroxyl) or binding to ions such as iron and copper which promote oxidative damage [29]. Furthermore, the main intracellular antioxidants are superoxide dismutase (SOD), glutathione (GSH) and catalase (CAT) [30,31]. Nitric oxide (NO), a free radical messenger molecule, is produced via the action of nitric oxide synthase on the L-arginine. Low levels of nitric oxide are thought to be physiological and protective, whereas its high levels as proinflammatory and injurious [32]. Recently, in CD patients, the overexpression of ROS and NO, as well as lower concentrations of numerous antioxidants, have been studied in intestinal mucosa or blood but not in saliva [31,32,33,34,35,36,37,38,39].

The study by Jahanshahi et al. [18] was the first evaluation of salivary oxidative and nitrosative stress in IBD patients. The antioxidant capacity of saliva was determined by measuring its ability to reduce Fe^3+^ to Fe^2+^ (FRAP—ferric reducing antioxidant power). Lipid peroxidation in samples was assessed based on the concentrations of adducts containing malondialdehyde (MDA) with 2-thiobarbituric acid (TBA). Levels of MDA, as the end product of the oxidation of polyunsaturated fatty acids, allow establishing the lipid peroxidation extent; while NO was assayed on the basis of the enzymatic conversion of nitrate to nitrite by nitrate reductase. Antioxidant power in the saliva of CD patients was significantly lower compared to healthy subjects. In addition, salivary concentrations of MDA increased significantly in this group. In contrast, salivary levels of FRAP and the products of lipid peroxidation were normal in UC patients. Explaining these surprising findings, the authors proposed that UC may not affect salivary glands and buccal mucosa. In CD several organs, including salivary glands, are oxidatively stressed as a result of chronic inflammation. However, the analysis of nitrosative stress showed a significant increment of NO in the saliva of both CD and UC patients in comparison to the control group. It is the first study determining increased salivary NO levels in IBD patients and supporting the theory that NO production may be an etiologic factor of these diseases as could cause epithelial and mucosal injuries. Moreover, researchers determined the salivary concentrations of epidermal growth factor (EGF). This main member of the EGF family is produced by submandibular glands and Brunner’s glands in the duodenum [40]. EGF stimulates the proliferation of epithelial and nonepithelial cells present in the gastrointestinal tract. It is also a stimulator of the expression of brush border enzymes and intestinal electrolyte and nutrient transport in enterocytes, as well as a promotor of intestinal healing [41,42]. In this study, CD patients showed compensated high concentrations of EGF and UC patients not depleted. The reason for these findings is unclear; the authors suggested that the altered levels of other EGF-family peptides, sharing the same receptor EGFR, may interfere.

In the year 2006, Rezaie et al. [21] conducted the study investigating alterations in salivary antioxidants, nitric oxide and transforming growth factor β1 (TGF-β1) in relation to disease activity in CD patients. A year later, the same authors [22] published a similar study considering the correlations between salivary nitric oxide and TGF-β1 and disease activity index, however, in UC patients. Only in the first study, salivary antioxidants, such as uric acid, albumin, transferrin and thiol groups (e.g., GSH), were determined. In addition, the researchers assessed the total antioxidant capacity by measuring FRAP and lipid peroxidation by MDA concentrations. Total NO level was assayed in the same way as in the previous study. In CD patients, the salivary levels of FRAP, uric acid and albumin were significantly reduced, as well as salivary transferrin and thiol groups but not significantly. MDA and NO concentrations were increased, respectively, five- and four-fold in comparison to the healthy subjects. No relationship was found between oxidative stress markers and NO. The disease activity was assessed according to Crohn’s Disease Activity Index (CDAI), including the number of liquid stools, the severity of abdominal pain, general well-being, extraintestinal manifestations (e.g., arthralgia, aphthous ulcers, anal fissures, fistulae or abscesses), abdominal mass, use of antidiarrheal drugs, haematocrit and body mass. CDAI significantly correlated with MDA, FRAP and the interaction between them (*r* = 0.8, *p* < 0.00005). In the constructed model, patients with a normal or low total antioxidant capacity had the lowest and the highest disease activity, depending on the MDA concentrations, whereas patients with high FRAP and high MDA levels had moderate disease activity due to the active defence of the organism against oxygen radicals. In turn, UC patients demonstrated an approximately four-fold increase in NO levels, without dependence on the activity of the disease. This finding was conflicting with previous studies concerning UC severity and NO levels in regions different to the oral cavity. The authors found that salivary oxidative stress could play a role in the pathogenesis of the CD (but not UC), modifying its course, and recommended further investigations. The results on salivary TGF-β1 from both studies will be discussed in the next section about inflammatory markers.

In the study from the year 2018, Szczeklik et al. [26] investigated the diagnostic usefulness of selected oxidative stress markers in the saliva and serum in CD patients (with an active and inactive form of the disease). Among the exclusion criteria, apart from smoking, diabetes, periodontal disease and using antioxidants in the past 6 months, were pregnancy or lactation, alcohol abuse, severe systemic disease, acute illness, oral mucosal disease, orthodontic appliances, current biologic therapy, abdominal abscess, intestinal stricture and active gastrointestinal bleeding. They determined levels of MDA (based on TBA reactivity), total antioxidant capacity (using FRAP), GSH (using the Ellman method) and CAT. In the routine laboratory tests, the haemoglobin concentration was lower, as well as the platelet count and serum C-reactive protein (CRP) level were higher in the patients with active CD than those with inactive and controls (*p* < 0.05). Serum and salivary MDA concentrations were increased, and the GSH levels decreased in active CD patients compared to inactive CD patients and controls. Levels of MDA in the saliva were higher than in the serum. Salivary FRAP levels were lower in CD patients, but not significantly, and there were no differences depending on the disease activity. The CAT activity was reduced in active CD patients in comparison to other groups, while in the earlier investigation, the authors reported a similar tendency for the SOD activity [43]. In the present study, a strong positive correlation between the serum or salivary MDA concentrations and CDAI values (both *r* = 0.8, *p* < 0.001) was observed. In addition, negative correlations between FRAP or GSH levels in the saliva and CDAI values were detected (*r* = −0.4, *p* = 0.04 and *r* = −0.05, *p* = 0.01, respectively). Moreover, there were significant positive correlations between the serum or salivary MDA concentrations with the CRP level (*r* = 0.6, *p* < 0.001 and *r* = 0.7, *p* < 0.001, respectively) and the platelet count (*r* = 0.6, *p* < 0.001 and *r* = 0.6, *p* = 0.001, respectively), as well as the negative with the haemoglobin level (*r* = −0.6, *p* < 0.001 and *r* = −0.05, *p* = 0.01, respectively). These relationships were determined using the Spearman rank correlation coefficient. The above significant associations between the increased MDA levels and clinical symptoms of disease severity suggested that it could have a valuable indicator for early CD diagnosis. The diagnostic usefulness of oxidative stress indicators was calculated by receiver operating characteristic (ROC) curves. Currently, the CRP seems to be the best biochemical marker for assessing and monitoring CD activity; however, more useful than for its diagnosis. The ROC analysis showed the good utility of MDA and CRP in differentiating active from inactive CD according to CDAI values (AUC = 0.95, cut-off point = 3.82 and AUC = 0.85, cut-off point = 4.25, respectively). The authors proposed that salivary biomarkers, such as MDA, can be used to assess oxidative stress in CD patients, both with the active course and the clinical remission.

### 3.2. Inflammatory Cytokines

Studies concerning oral diseases, such as periodontal diseases, lichen planus or oral carcinoma, demonstrated the increased salivary levels of selected pro-inflammatory nuclear factor-κB dependent cytokines, e.g., interleukin 1β (IL-1β), interleukin 6 (IL-6), tumour necrosis factor α (TNF-α) [44,45,46]. They play an important role in the promotion of inflammatory processes. These cytokines have also been reported in the saliva of patients with several systemic disorders, including inter alia rheumatoid arthritis, psoriasis, lysosomal storage diseases, diabetes or obesity [47,48,49,50,51]. However, the salivary biomarkers of inflammatory processes in IBD patients have been rarely investigated. The limitation may be the fact that the IBD patients are chronically treated with anti-inflammatory drugs (such as aminosalicylates, thiopurines or glucocorticoids), affecting the measured cytokines.

The study of Nielsen et al. [20] aimed to investigate the inflammatory process using a non-invasive method for measuring IL-6 concentrations in the saliva from IBD patients. Other studies showed higher IL-6 levels in colonic biopsies of inflamed mucosa in IBD patients, however, in saliva it had not been reported previously [52,53]. IL-6 is produced mainly by macrophages and monocytes, and to a lesser extent by endothelial cells, fibroblasts and lymphocytes. This multifunctional cytokine is responsible for the regulation of the inflammatory processes, immune and acute-phase response, as well as the stimulation of haematopoiesis and thrombopoiesis [54]. In this study, the clinical disease severity was scored using CDAI in CD patients and the Seo clinical colitis activity index (AI) in UC patients. Participants with severe inflammation, acute infections, pregnancy, periodontal diseases and other chronic inflammatory diseases were excluded. Salivary IL-6 concentrations were significantly higher in CD patients but not significantly in UC patients. In contrast, a significant difference in plasma IL-6 levels was observed in both groups (*p* < 0.001). In UC patients, significant positive correlations between salivary IL-6 concentrations and plasma concentrations or AI score (*r*^2^ = 0.81, *p* < 0.01 and *r*^2^ = 0.61, *p* < 0.05, respectively) were demonstrated, as well as negative with albumin level (*r*^2^ = 0.83, *p* < 0.01). No similar relationships were found in the CD patients. IL-6 levels in the saliva of both groups were not associated with CRP levels. Authors suggested that salivary IL-6 may have prognostic value in the monitoring of IBD patients, especially CD patients.

In the other study from the year 2012, Szczeklik et al. [25] examined if salivary concentrations of selected proinflammatory cytokines (such as IL-1β, IL-6 and TNF-α) were associated with the activity and prevalence of oral lesions in CD patients. They excluded subjects with systemic infections, cardiovascular, pulmonary and kidney diseases, autoimmune diseases, diabetes, allergy, chronic periodontitis or oral lichen planus and treated with anti-inflammatory drugs (except azathioprine), antioxidants or statins. The severity of CD was classified according to CDAI—low 150–220 and moderate 220–450. As expected, in active CD patients significantly lower body mass index (BMI), red blood cell count (RBC) and haemoglobin concentration (*p* < 0.01), as well as higher platelet count and CRP level (*p* < 0.001), were observed compared to inactive CD patients and controls. The salivary flow rate did not differ between these groups or correlate with the prevalence of oral manifestations in both CD groups. The ELISA analysis demonstrated significantly elevated salivary levels of IL-1β, IL-6 and TNF-α in active CD patients. No significant differences were found between inactive CD patients and controls. In the Spearman rank test, positive correlations between higher salivary concentrations of IL-6 or TNF-α and the prevalence of specific oral lesions in active CD were observed, while no such association was seen for IL-1β. The researchers speculated that the reason for this finding might be the antagonistic influence of CD drugs on salivary IL-1β. In addition, the present study showed a relationship between oral manifestations and standard laboratory parameters (such as RBC, haemoglobin, platelets, CRP), however, not with CDAI values. Authors concluded that active CD patients had altered cytokine production reflected by increased concentrations of proinflammatory cytokines in the saliva. Salivary cytokine levels may be sensitive biomarkers of CD activity.

TGF-β1 plays an essential anti-inflammatory role by counteracting TNF-α [55,56,57]. As mentioned earlier, Rezaie et al. [21,22] investigated TGF-β1 levels in IBD patients. Although TGF-β1 concentrations were significantly increased in CD patients compared to control subjects, they had no correlation with CDAI or oxidative stress markers. Moreover, in UC patients, they observed significantly enhanced salivary TGF-β1 levels, without any relationship with disease activity. It was explained that increased levels of TGF-β1 are associated with the defence stimulation of the differentiation of epithelial cells and the promotion of damaged mucosa repair.

Study of Said et al. [23] presented that oral dysbiosis was strongly associated with elevated inflammatory cytokines and lowered lysozyme in the saliva of IBD patients. The used Luminex technology allows measuring cytokines from little volumes of saliva samples with high sensitivity. Salivary concentrations of IL-6, IL-8 and MCP-1 were significantly higher in UC patients, while TNF-α was in CD patients. Additionally, in the saliva of both groups significantly elevated levels of IL-1β, IgA and LL37 (cathelicidin) were found. Cathelicidins are produced by the epithelial cells of mucosa in response to invasive bacterial infection [58]. In contrast, the salivary lysozyme level was significantly reduced compared to the healthy subjects. This antimicrobial protein (produced by macrophages, neutrophils and epithelial cells) plays a key role in the host constitutive defence system [59]. The lysozyme hydrolyses the cell wall of Gram-positive bacteria and may kill Gram-negative bacteria through synergic action with salivary lactoferrin [60]. Previous studies reported elevated levels of faecal lysozyme in IBD patients [61]. The alterations in salivary inflammatory biomarkers were strongly correlated with the abundance of four bacterial genera: *Streptococcus*, *Prevotella*, *Veillonella* and *Haemophilus*. The authors postulated that salivary microbiota could affect the gastrointestinal microbiota to some extent.

### 3.3. MicroRNAs

MicroRNAs (miRNAs) are single-stranded RNA molecules, consisting of 19–25 nucleotides. These non-coding RNAs are involved in the regulation of gene expression post-transcriptionally to stop translation and promote mRNA degradation. Over 30% of the genome is predicted to be regulated by miRNAs [62]. Previous studies showed that miRNAs are responsible for various cellular processes, including differentiation, proliferation, apoptosis, metabolism, and oncogenesis [63,64]. Thereby, the aberrant expression of miRNAs has been associated with a growing amount of disorders, such as cancer and autoimmune diseases [65,66,67]. Several studies determined miRNAs related to IBD in intestinal biopsies and blood [68,69,70,71,72].

In this review, we present the only study considering salivary miRNAs in IBD patients to date. Schaefer et al. [24] identified specific miRNAs which could discriminate CD from UC and controls using colon, blood and saliva specimens. Saliva seems to be an ideal fluid for the multiple monitoring of disease progression because it provides a more accurate “real-time read-out” than serum. In saliva samples, miR-101 was significantly overexpressed in CD patients, as well as miR-21, miR-31 and miR-142-3p in UC patients compared to the control group. In addition, miR-142-5p was significantly underexpressed in UC patients relative to healthy subjects. miR-21 is associated with inflammatory and oncological diseases [73,74,75]. miR-31 is described as an oncomiR in lung cancer and a tumour suppressor in breast cancer [76,77]. miR-101 has anti-proliferative properties; it is reported to be a negative regulator of an inducible costimulator (ICOS) in inflammation processes and a negative regulator of corepressor C-terminal binding protein-2 (CtBP2) [78,79]. Authors indicated that miR-101 could be an essential regulator in IBD because it was significantly elevated in all three tissues of CD patients and two of three in UC patients. Moreover, it was suggested that extraintestinal fluids might effectively reflect the intestinal inflammation without the need for a colon biopsy. Saliva and blood as diagnostic materials can support early detection techniques for IBD. It was a first study reporting altered miRNA expression in saliva, so further investigations are necessary.

### 3.4. Other Biomarkers

Exosomes are small spherical vesicles with a diameter of 40–120 nm [80]. They may contain proteins (including enzymes), lipids and RNAs (including miRNAs) [81,82]. Exosomes perform multiple functions, such as the distribution of cell substances, immune regulation and the propagation of prion protein or retroviruses [83]. In the year 2013, the Nobel Prize in Physiology or Medicine was awarded jointly to James E. Rothman, Randy W. Schekman and Thomas C. Südhof “for their discoveries of machinery regulating vesicle traffic, a major transport system in our cells”. Due to their stability exosomes can move far away without rupture. They exist in almost every type of cell and biological fluid, including saliva [84]. Zheng et al. [28] explored a new exosomal biomarker in the saliva of IBD patients. After screening various proteins in salivary exosomes, they chose the eight proteins present only in every IBD patient. Among these proteins related to inflammation and immune response, proteasome subunit alpha type-7 (PSMA7) was distributed in the most Gene Ontology (GO) terms with a high enrichment score. The Western blotting showed significantly elevated levels of PSMA7 in IBD patients relative to the controls. Patients in remission demonstrated decreased exosomal PSMA7 levels compared to patients with an active course. Previous studies reported that increased PSMA7 expression was associated with the liver metastasis of colon cancer or depressive disorders [85,86]. Authors believed that exosomal PSMA7 in the saliva is a useful biomarker for the development of IBD. However, due to the ability of exosomes for long “wandering”, it is impossible to be sure that some of them detected in the saliva did not originate from other cells, such as intestinal.

The next diagnostic biomarker can be salivary α-amylase (sAA) described by Xu et al. [27] in UC patients. According to the literature, this protein’s production positively correlates with the copy number of AMY1 gene [87,88]. Salivary α-amylase secretion is mainly regulated by the sympathetic nervous system—released noradrenaline binds to the β-adrenergic receptor on the acinar cells of the salivary glands, inducing an increase in the intracellular cyclic adenosine monophosphate followed by sAA secretion from these cells. In contrast, the salivary flow rate is mostly controlled by the parasympathetic nervous system—released acetylcholine binds to the M3 muscarinic receptor on the acinar cells, stimulating elevation of intracellular calcium and subsequently saliva secretion [89,90,91]. Recently, salivary α-amylase has been reported as a non-invasive indicator for the sympathetic nervous activity [92]. In this study, significantly elevated sAA concentrations were detected in UC patients compared to healthy subjects both before and after gustatory stimulation. However, no significant differences in the unstimulated and stimulated salivary flow rates were found between the UC patients and controls. These findings could implicate the sympathetic overactivity in the pathogenesis of UC. In addition, previous studies suggested that the sympathetic nervous activity was associated with mediating intestinal inflammation in this disease, while the parasympathetic nervous activity might exhibit anti-inflammatory properties [93,94,95].

The newest included study from the year 2019, conducted by Majster et al. [19], assessed the concentration of calprotectin in the unstimulated and stimulated saliva of active IBD patients before and after treatment. Calprotectin (also named migration inhibitory factor-related protein 8/14 or S100A8/A9) is a calcium-binding antimicrobial protein complex. It is produced by neutrophils, monocytes, macrophages and keratinocytes [96]. This acute-phase protein has many intra- and extracellular functions, such as the regulation of cytoskeleton–plasma interactions and the homeostasis of neutrophils, as well as the promotion of inflammation (e.g., through the endogenous activation of Toll-like receptor-4 or receptor for advanced glycation end-products) [97,98,99,100]. To date, faecal calprotectin has been used as a diagnostic marker for the detection and monitoring of IBD [101,102]. Calprotectin has also been reported in the saliva of patients with periodontitis or Sjögren’s syndrome [103,104]. In the present study, in stimulated saliva, calprotectin levels were significantly higher than in the unstimulated saliva. Moreover, fasting and gender did not alter the calprotectin concentrations. In CD patients, significantly elevated levels of calprotectin were determined in both unstimulated and stimulated saliva in comparison to the controls. However, in UC patients, significantly increased calprotectin concentrations were observed only in stimulated saliva. In addition, CD patients tended to have higher levels of calprotectin in stimulated saliva relative to UC patients (*p* = 0.065). There were no significant differences in the salivary calprotectin concentrations before and after 10–12 weeks of treatment (*p* = 0.140 for unstimulated and *p* = 0.650 for stimulated). In contrast, the median levels of serum calprotectin were 3.3-fold lower at the follow-up compared to baseline (*p* = 0.011). The authors found that salivary calprotectin could discriminate IBD patients from controls based on ROC analysis. For the unstimulated saliva AUC was 0.927 and for stimulated −0.870. This study had several limitations, such as a lack of oral examination and differences in nicotine consumption between groups. Further studies enclosing standardised disease activity scores are advisable.

## 4. Materials and Methods 

### 4.1. Search Strategy and Data Extraction

This systematic review was conducted up to 28 June 2020, according to the Preferred Reporting Items for Systematic Reviews and Meta-Analyses (PRISMA) statement guidelines [105], using the databases PubMed, Scopus and Web of Science. The search formula included “saliva” and “inflammatory bowel disease” as MeSH (medical subject headings) terms combined in PubMed Advanced Search Builder. In other databases combinations of the keywords—“saliva*” and “inflammatory bowel disease*” or “IBD” or “ulcerative colitis” or “Crohn’s disease”—were used (in Scopus as indexterms and in Web of Science as author keywords or keywords plus).

Records were screened by the title, abstract and full text by two independent investigators. Studies included in this review matched all the predefined criteria according to the PICOS (“population”, “intervention”, “comparison”, “outcomes”, “study design”)—Table 4. The detailed search flowchart is presented in Figure 1 (in the section Results). 

### 4.2. Quality Assessment and Critical Appraisal

**Figure 2:**
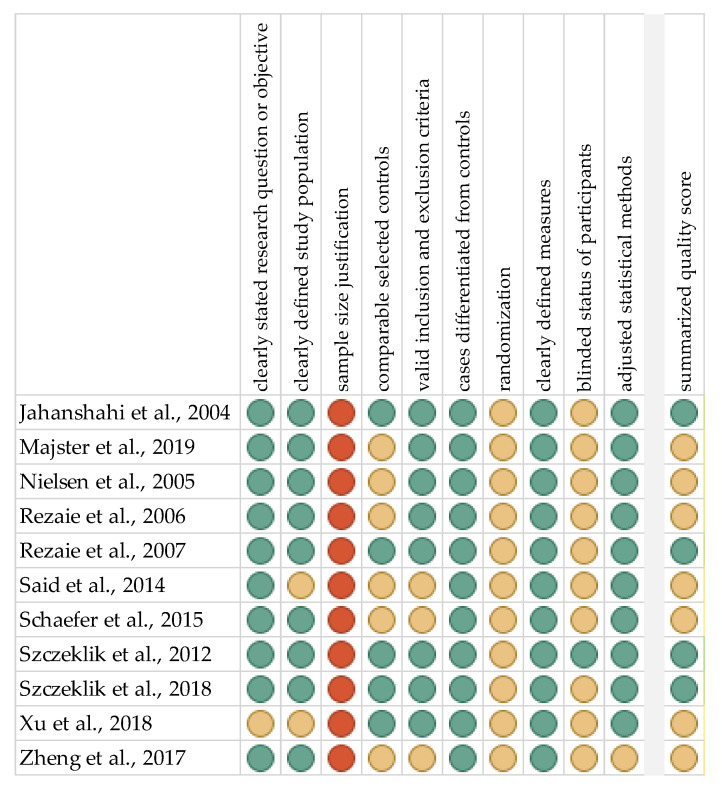
Quality assessment, including the main potential risk of bias (risk level: green—low, yellow—unspecified, red—high; quality score: green—good, yellow—intermediate, red—poor).

The risk of bias in each individual study was assessed according to the “Study Quality Assessment Tool” issued by the National Heart, Lung, and Blood Institute within the National Institute of Health [106]. These questionnaires were answered by two independent investigators, and disagreements were resolved by discussion between them. The summarised quality assessment for every single study is reported in Figure 2. The most frequently encountered risks of bias were the absence of data regarding sample size justification (all studies), randomisation (all studies) and blinding (ten studies). Critical appraisal was summarised through adding up the points for each criterion of potential risk (points: 1—low, 0.5—unspecified, 0—high). Four studies (36.4%) were classified as having a “good” quality (≥80% total score) and seven (63.6%) as “intermediate” (≥60% total score).

The level of evidence was assessed using the classification of the Oxford Centre for Evidence-Based Medicine levels for diagnosis [107]. All of the included studies have a low fourth level of evidence (in this 5-graded scale), because of their case-control design.

## 5. Conclusions

In conclusion, according to our systematic review, saliva contains several biomarkers (e.g., proteins or miRNAs) which can be used credibly to detect and control patients with Crohn’s disease or ulcerative colitis. However, further investigations are necessary to validate these findings, as well as to identify new reliable salivary biomarkers for the early diagnosis and regular monitoring of inflammatory bowel diseases.

## Figures and Tables

**Table 1:** General characteristics of included studies.

Author, Year, Setting	IBD Patients (F/M)	Control Patients (F/M)	Pharmacological Treatment	Smoking Habits	Salivary Biomarkers
Jahanshahi et al., 2004, Iran [18]	16 (8/8) CD, 16 (8/8) UC	16 (8/8)	NR	Non-smokers	CD: MDA, EGF, NO, FRAP↓; UC: NO
Majster et al., 2019, Sweden [19]	23 (9/14): 12 CD, 11 UC	15 (9/6)	Glucocorticoids 13; anti-TNF/biologicals 6; Thiopurines 5; 5-ASA 3; None 2	8 smokers, 6 former smokers, 9 non-smokers; 15 control non-smokers	Calprotectin
Nielsen et al., 2005, Denmark [20]	15 (10/5) CD, 7 (4/3) UC	19 (15/4)	5-ASA 10/6 (CD/UC); Prednisolone 2/0; Budesonide 1/2; Azathioprine 4/0; None 2/1	NR	IL-6
Rezaie et al., 2006, Iran [21]	28 (17/11) CD	20 (8/12)	5-ASA 28; Corticosteroids 6; Azathioprine 10	Non-smokers	TGF-β1, NO, MDA, FRAP↓, albumin↓, uric acid↓
Rezaie et al., 2007, Iran [22]	37 (23/14) UC	15 (sex-matched)	5-ASA 36; Corticosteroids 14; Azathioprine 13	Non-smokers	TGF-β1, NO
Said et al., 2014, Japan [23]	14 CD, 10 UC	15	NR	NR	IL-1β, lysozyme↓, IgA, LL37; CD: TNF-α; UC: IL-6, IL-8, MCP-1
Schaefer et al., 2015, USA [24]	5 CD, 5 UC	5	NR	NR	CD: miR-101; UC: miR-21, miR-31, miR-142-3p, miR-142-5p↓
Szczeklik et al., 2012, Poland [25]	52 (16/36) active CD, 43 (14/29) inactive CD	45 (17/28)	Mesalazine and azathioprine	Smokers: 14 active, 13 inactive, 12 controls	IL-1β, IL-6, TNF-α
Szczeklik et al., 2018, Poland [26]	32 (13/19) active CD, 26 (9/17) inactive CD	26 (12/14)	Mesalazine and azathioprine	Non-smokers	MDA, GSH↓, CAT↓
Xu et al., 2018, China [27]	35 (18/17) UC	32 (15/17)	NR	NR	α-amylase
Zheng et al., 2017, China [28]	11 CD, 37 UC	10 (sex-matched)	NR	NR	PSMA7 (exosomal)

Legend: CD, Crohn’s disease; UC, ulcerative colitis; F, females; M, males; NR, not reported; 5-ASA, 5-aminosalicylic acid; MDA, malondialdehyde; EGF, epidermal growth factor; NO, nitric oxide; FRAP, ferric reducing antioxidant power; IL, interleukin; TGF-β1, transforming growth factor β1; TNF-α, tumour necrosis factor α; MCP-1, monocyte chemoattractant protein-1; miR, microRNA; GSH, glutathione; CAT, catalase; PSMA7, proteasome subunit alpha type-7; ↓, decreased level.

**Table 2:** Methods of collection and analysis of saliva.

Study	Type of Saliva and Method of Collection	Centrifugation and Storing	Methods of Analysis	Salivary Biomarkers
Jahanshahi et al., 2004 [18]	Unstimulated whole saliva 3ml	Centrifuged at 10,000× *g* for 5 min, stored at −80 °C until analysis	Fluorescence spectrophotometry	MDA, FRAP
			ELISA	EGF, NO
Majster et al., 2019 [19]	Unstimulated whole saliva and stimulated whole saliva (through chewing on a 0.5 g paraffin tablet for 5 min); in controls under fasting and non-fasting conditions	Immediately placed on ice, centrifuged (NR parameters), stored at −80°C until analysis	ELISA	Calprotectin
Nielsen et al., 2005 [20]	Unstimulated whole saliva for 2–5 min; 4 times with a 1 h interval between collection periods; not to eat, drink, smoke, brush the teeth or chew gum 15 min before saliva collections	Immediately stored at −20 °C, then completed 4 samples stored at −80 °C until analysis, before analysis centrifuged at 3000× *g* for 10 min	ELISA	IL-6
Rezaie et al., 2006 [21]	Unstimulated whole saliva for 5 min	Centrifuged at 10,000× *g* for 5 min, stored at −80 °C until analysis	Fluorescence spectrophotometry	MDA; FRAP, albumin, uric acid
			ELISA	TGF-β1, NO
Rezaie et al., 2007 [22]	Unstimulated whole saliva 3ml	Centrifuged at 10,000× *g* for 5 min, stored at −80 °C until analysis	ELISA	TGF-β1, NO
Said et al., 2014 [23]	Unstimulated whole saliva	Immediately frozen by liquid nitrogen, stored at −80 °C until use	Fluorescence technique	IL-1β, IL-6, IL-8, TNF-α, MCP-1
			ELISA	LL37
			EIA	IgA
			Turbidimetric technique	Lysozyme
Schaefer et al., 2015 [24]	Unstimulated whole saliva	NR	qRT-PCR	miR-101, miR-21, miR-31, miR-142-3p, miR-142-5p
Szczeklik et al., 2012 [25]	Unstimulated whole saliva for 15 min on ice; between 8 and 10 a.m. in fasting patients	Centrifuged at 3500 rpm for 20 min, stored at −80 °C until analysis	ELISA	IL-1β, IL-6, TNF-α
Szczeklik et al., 2018 [26]	Unstimulated whole saliva collected in precooled tubes; between 8 and 10 a.m. in fasting patients	Centrifuged at 1000× *g* for 10 min at 4 °C, stored at −80 °C until analysis	Fluorescence spectrophotometry	MDA, GSH, CAT
Xu et al., 2018 [27]	Whole saliva unstimulated and stimulated after citric acid; in the morning; not to eat or drink (except water) or do exercise before collection	Centrifuged at 12,000× *g* for 15 min	Western blotting	α-amylase
Zheng et al., 2017 [28]	Unstimulated whole saliva 5ml; not to eat or drink after dinner the previous evening or to brush teeth on the collection day morning	Kept on ice, centrifuged at 10,000× *g* for 10 min at 4 °C	Western blotting	PSMA7 (exosomal)

Legend: NR, not reported; ELISA, enzyme-linked immunosorbent assay; EIA, enzyme immunoassay; qRT-PCR, quantitative reverse transcriptase polymerase chain reaction; MDA, malondialdehyde; EGF, epidermal growth factor; NO, nitric oxide; FRAP, ferric reducing antioxidant power; IL, interleukin; TGF-β1, transforming growth factor β1; TNF-α, tumour necrosis factor α; MCP-1, monocyte chemoattractant protein-1; miR, microRNA; GSH, glutathione; CAT, catalase; PSMA7, proteasome subunit alpha type-7.

**Table 3:** Statistical significance for salivary biomarkers in IBD.

Study	Salivary Biomarkers *	*p*-Value		AUC-ROC
Jahanshahi et al., 2004 [18]	CD: MDA, EGF, NO, FRAP↓	<0.01		
	UC: NO			
Majster et al., 2019 [19]	Calprotectin	CD: 0.011 (unstimulated)		Unstimulated: 0.927 (95% CI, 0.838–1.000)
		UC: 0.076 (unstimulated)		
		CD: 0.002 (stimulated)		Stimulated: 0.870 (95% CI, 0.752–0.987)
		UC: 0.021 (stimulated)		
Nielsen et al., 2005 [20]	IL-6	CD: <0.05,		
		UC: 0.09		
Rezaie et al., 2006 [21]	TGF-β1	0.03		
	NO	<0.00005		
	MDA			
	FRAP↓	0.02		
	Albumin↓	0.01		
	Uric acid↓	0.03		
Rezaie et al., 2007 [22]	TGF-β1	0.005		
	NO	<0.00005		
Said et al., 2014 [23]	IL-1β	CD: <0.05 UC: <0.01		
	Lysozyme↓	<0.01		
	IgA, LL37	<α (NR)		
	TNF-α	CD: <α		
	IL-6, IL-8, MCP-1	UC: <α		
Schaefer et al., 2015 [24]	CD: miR-101	<0.05		
	UC: miR-21, miR-31, miR-142-3p, miR-142-5p↓			
Szczeklik et al., 2012 [25]	IL-1β	active vs. controls <0.01	active vs. inactive <0.039	
	IL-6	<0.01	<0.041	
	TNF-α	<0.001	<0.002	
Szczeklik et al., 2018 [26]	MDA	0.01		0.95 (95% CI, 0.90–1.00)
	GSH↓	0.01		
	CAT↓	0.001		
Xu et al., 2018 [27]	α-amylase	0.015 (unstimulated)		
		0.021 (stimulated)		
Zheng et al., 2017 [28]	PSMA7 (exosomal)	NR		

Legend: * elevated concentrations, expect these marked with ↓; CD, Crohn’s disease; UC, ulcerative colitis; MDA, malondialdehyde; EGF, epidermal growth factor; NO, nitric oxide; FRAP, ferric reducing antioxidant power; IL, interleukin; TGF-β1, transforming growth factor β1; TNF-α, tumour necrosis factor α; MCP-1, monocyte chemoattractant protein-1; miR, microRNA; GSH, glutathione; CAT, catalase; PSMA7, proteasome subunit alpha type-7; NR, not reported; α, significance level; AUC-ROC, area under curve-receiver operating characteristic; CI, confidence interval.

**Table 4:** Inclusion and exclusion criteria according to the PICOS.

Parameter	Inclusion Criteria	Exclusion Criteria
Population	Patients with IBD (Crohn’s disease & ulcerative colitis)—aged from 0 to 99 years, both sexes	Patients with another bowel disease or autoimmune disease
Intervention	Not applicable	
Comparison	Not applicable	
Outcomes	Salivary biomarkers diagnostic for IBD (proteins, miRNAs etc.)	Changes in salivary microbiota
Study design	Case-control, cohort and cross-sectional studies	Literature reviews, case reports, expert opinion, letters to editor, conference reports
	Published after 2000	Not published in English

The heterogeneity of the salivary biomarkers, as well as of the diseases (Crohn’s disease and ulcerative colitis), did not allow to perform a meta-analysis of the studies included in the present systematic review.

## Abbreviations

IBD	inflammatory bowel disease
CD	Crohn’s disease
UC	ulcerative colitis
NR	not reported
5-ASA	5-aminosalicylic acid
ELISA	enzyme-linked immunosorbent assay
EIA	enzyme immunoassay
qRT-PCR	quantitative reverse transcriptase polymerase chain reaction
AUC-ROC	area under curve-receiver operating characteristic
ROS	reactive oxygen species
SOD	superoxide dismutase
GSH	glutathione
CAT	catalase
NO	nitric oxide
FRAP	ferric reducing antioxidant power
MDA	malondialdehyde
TBA	2-thiobarbituric acid
EGF	epidermal growth factor
TGF-β1	transforming growth factor β1
CDAI	Crohn’s disease activity index
CRP	C-reactive protein
IL-1β	interleukin 1
IL-6	interleukin 6
TNF-α	tumour necrosis factor α
MCP-1	monocyte chemoattractant protein-1
miR	microRNA
PSMA7	proteasome subunit alpha type-7
sAA	salivary α-amylase

## References

[1] Rehak N.N., Cecco S.A., Csako G. (2000). Biochemical composition and electrolyte balance of “unstimulated” whole human saliva. Clin. Chem. Lab. Med..

[2] Lee Y.-H., Wong D.T. (2009). Saliva: An emerging biofluid for early detection of diseases. Am. J. Dent..

[3] Segal A., Wong D.T. (2008). Salivary diagnostics: Enhancing disease detection and making medicine better. Eur. J. Dent. Educ..

[4] Denny P., Hagen F.K., Hardt M., Liao L., Yan W., Arellanno M., Bassilian S., Bedi G.S., Boontheung P., Cociorva D. (2008). The proteomes of human parotid and submandibular/sublingual gland salivas collected as the ductal secretions. J. Proteome Res..

[5] Li Y., Zhou X., St John M.A.R., Wong D.T.W. (2004). RNA profiling of cell-free saliva using microarray technology. J. Dent. Res..

[6] Park N.J., Zhou H., Elashoff D., Henson B.S., Kastratovic D.A., Abemayor E., Wong D.T. (2009). Salivary microRNA: Discovery, characterization, and clinical utility for oral cancer detection. Clin. Cancer Res..

[7] Yeh C.-K., Christodoulides N.J., Floriano P.N., Miller C.S., Ebersole J.L., Weigum S.E., McDevitt J., Redding S.W. (2010). Current development of saliva/oral fluid-based diagnostics. Tex. Dent. J..

[8] Nijakowski K., Surdacka A. (2018). Saliva as a biological fluid in diagnostics of systemic diseases—A literature review. Dental Forum.

[9] Vitorino R., de Morais Guedes S., Ferreira R., Lobo M.J.C., Duarte J., Ferrer-Correia A.J., Tomer K.B., Domingues P.M., Amado F.M.L. (2006). Two-dimensional electrophoresis study of in vitro pellicle formation and dental caries susceptibility. Eur. J. Oral Sci..

[10] Rudney J.D., Staikov R.K., Johnson J.D. (2009). Potential biomarkers of human salivary function: A modified proteomic approach. Arch. Oral Biol..

[11] Fábián T.K., Fejérdy P., Csermely P. (2008). Salivary genomics, transcriptomics and proteomics: The emerging concept of the oral ecosystem and their use in the early diagnosis of cancer and other diseases. Curr. Genomics.

[12] Kibayashi M., Tanaka M., Nishida N., Kuboniwa M., Kataoka K., Nagata H., Nakayama K., Morimoto K., Shizukuishi S. (2007). Longitudinal study of the association between smoking as a periodontitis risk and salivary biomarkers related to periodontitis. J. Periodontol..

[13] Nishida N., Yamamoto Y., Tanaka M., Maeda K., Kataoka K., Nakayama K., Morimoto K., Shizukuishi S. (2006). Association between passive smoking and salivary markers related to periodontitis. J. Clin. Periodontol..

[14] Kaplan G.G. (2015). The global burden of IBD: From 2015 to 2025. Nat. Rev. Gastroenterol. Hepatol..

[15] Kmieć Z. (1998). Cytokines in inflammatory bowel disease. Arch. Immunol. Ther. Exp. (Warsz.).

[16] Dignass A., Van Assche G., Lindsay J.O., Lémann M., Söderholm J., Colombel J.F., Danese S., D’Hoore A., Gassull M., Gomollón F. (2010). The second European evidence-based Consensus on the diagnosis and management of Crohn’s disease: Current management. J. Crohns Colitis.

[17] Muhvić-Urek M., Tomac-Stojmenović M., Mijandrušić-Sinčić B. (2016). Oral pathology in inflammatory bowel disease. World J. Gastroenterol..

[18] Jahanshahi G., Motavasel V., Rezaie A., Hashtroudi A.A., Daryani N.E., Abdollahi M. (2004). Alterations in antioxidant power and levels of epidermal growth factor and nitric oxide in saliva of patients with inflammatory bowel diseases. Dig. Dis. Sci..

[19] Majster M., Almer S., Boström E.A. (2019). Salivary calprotectin is elevated in patients with active inflammatory bowel disease. Arch. Oral Biol..

[20] Nielsen A.A., Nielsen J.N., Schmedes A., Brandslund I., Hey H. (2005). Saliva interleukin-6 in patients with inflammatory bowel disease. Scand. J. Gastroenterol..

[21] Rezaie A., Ghorbani F., Eshghtork A., Zamani M.J., Dehghan G., Taghavi B., Nikfar S., Mohammadirad A., Daryani N.E., Abdollahi M. (2006). Alterations in salivary antioxidants, nitric oxide, and transforming growth factor-beta 1 in relation to disease activity in Crohn’s disease patients. Ann. N. Y. Acad. Sci..

[22] Rezaie A., Khalaj S., Shabihkhani M., Nikfar S., Zamani M.J., Mohammadirad A., Daryani N.E., Abdollahi M. (2007). Study on the correlations among disease activity index and salivary transforming growth factor-beta 1 and nitric oxide in ulcerative colitis patients. Ann. N. Y. Acad. Sci..

[23] Said H.S., Suda W., Nakagome S., Chinen H., Oshima K., Kim S., Kimura R., Iraha A., Ishida H., Fujita J. (2014). Dysbiosis of salivary microbiota in inflammatory bowel disease and its association with oral immunological biomarkers. DNA Res..

[24] Schaefer J.S., Attumi T., Opekun A.R., Abraham B., Hou J., Shelby H., Graham D.Y., Streckfus C., Klein J.R. (2015). MicroRNA signatures differentiate Crohn’s disease from ulcerative colitis. BMC Immunol..

[25] Szczeklik K., Owczarek D., Pytko-Polończyk J., Kęsek B., Mach T.H. (2012). Proinflammatory cytokines in the saliva of patients with active and non-active Crohn’s disease. Pol. Arch. Intern. Med..

[26] Szczeklik K., Krzyściak W., Cibor D., Domagała-Rodacka R., Pytko-Polończyk J., Mach T., Owczarek D. (2018). Markers of lipid peroxidation and antioxidant status in the serum and saliva of patients with active Crohn disease. Pol. Arch. Intern. Med..

[27] Xu Z., Wei B., Qiu Y., Zhang T. (2018). Altered salivary alpha-amylase secretion in patients with ulcerative colitis. Gastroenterol. Res. Pract..

[28] Zheng X., Chen F., Zhang Q., Liu Y., You P., Sun S., Lin J., Chen N. (2017). Salivary exosomal PSMA7: A promising biomarker of inflammatory bowel disease. Protein Cell.

[29] Moore S., Calder K.A., Miller N.J., Rice-Evans C.A. (1994). Antioxidant activity of saliva and periodontal disease. Free Radic. Res..

[30] Alzoghaibi M.A., Al-Mofleh I.A., Al-Jebreen A.M. (2014). Antioxidant activities for superoxide dismutase in patients with Crohn’s disease. J. Basic Clin. Physiol. Pharmacol..

[31] Kruidenier L., Kuiper I., Lamers C.B.H.W., Verspaget H.W. (2003). Intestinal oxidative damage in inflammatory bowel disease: Semi-quantification, localization, and association with mucosal antioxidants. J. Pathol..

[32] Cross R.K., Wilson K.T. (2003). Nitric oxide in inflammatory bowel disease. Inflamm. Bowel Dis..

[33] Lih-Brody L., Powell S.R., Collier K.P., Reddy G.M., Cerchia R., Kahn E., Weissman G.S., Katz S., Floyd R.A., McKinley M.J. (1996). Increased oxidative stress and decreased antioxidant defenses in mucosa of inflammatory bowel disease. Dig. Dis. Sci..

[34] Pinto M.A.S., Lopes M.S.-M.S., Bastos S.T.O., Reigada C.L.L., Dantas R.F., Neto J.C.B., Luna A.S., Madi K., Nunes T., Zaltman C. (2013). Does active Crohn’s disease have decreased intestinal antioxidant capacity?. J. Crohns Colitis.

[35] Mohammadi E., Qujeq D., Taheri H., Hajian-Tilaki K. (2017). Evaluation of serum trace element levels and superoxide dismutase activity in patients with inflammatory bowel disease: Translating Basic Research into Clinical Application. Biol. Trace Elem. Res..

[36] Rachmilewitz D., Stamler J.S., Bachwich D., Karmeli F., Ackerman Z., Podolsky D.K. (1995). Enhanced colonic nitric oxide generation and nitric oxide synthase activity in ulcerative colitis and Crohn’s disease. Gut.

[37] Wendland B.E., Aghdassi E., Tam C., Carrrier J., Steinhart A.H., Wolman S.L., Baron D., Allard J.P. (2001). Lipid peroxidation and plasma antioxidant micronutrients in Crohn disease. Am. J. Clin. Nutr..

[38] Ardite E., Sans M., Panés J., Romero F.J., Piqué J.M., Fernández-Checa J.C. (2000). Replenishment of glutathione levels improves mucosal function in experimental acute colitis. Lab. Invest..

[39] Moret I., Cerrillo E., Navarro-Puche A., Iborra M., Rausell F., Tortosa L., Beltrán B. (2014). Oxidative stress in Crohn’s disease. Gastroenterol. Hepatol..

[40] Playford R.J. (1995). Peptides and gastrointestinal mucosal integrity. Gut.

[41] Opleta-Madsen K., Hardin J., Gall D.G. (1991). Epidermal growth factor upregulates intestinal electrolyte and nutrient transport. Am. J. Physiol..

[42] Riegler M., Sedivy R., Sogukoglu T., Cosentini E., Bischof G., Teleky B., Feil W., Schiessel R., Hamilton G., Wenzl E. (1996). Epidermal growth factor promotes rapid response to epithelial injury in rabbit duodenum in vitro. Gastroenterology.

[43] Szczeklik K., Krzysciak W., Domagala-Rodacka R., Mach P., Darczuk D., Cibor D., Pytko-Polonczyk J., Rodacki T., Owczarek D. (2016). Alterations in glutathione peroxidase and superoxide dismutase activities in plasma and saliva in relation to disease activity in patients with Crohn’s disease. J. Physiol. Pharmacol..

[44] Rhodus N.L., Cheng B., Myers S., Bowles W., Ho V., Ondrey F. (2005). A comparison of the pro-inflammatory, NF-kappaB-dependent cytokines: TNF-alpha, IL-1-alpha, IL-6, and IL-8 in different oral fluids from oral lichen planus patients. Clin. Immunol..

[45] Teles R.P., Likhari V., Socransky S.S., Haffajee A.D. (2009). Salivary cytokine levels in subjects with chronic periodontitis and in periodontally healthy individuals: A cross-sectional study. J. Periodont. Res..

[46] Rhodus N.L., Ho V., Miller C.S., Myers S., Ondrey F. (2005). NF-kappaB dependent cytokine levels in saliva of patients with oral preneoplastic lesions and oral squamous cell carcinoma. Cancer Detect. Prev..

[47] Mirrielees J., Crofford L.J., Lin Y., Kryscio R.J., Dawson D.R., Ebersole J.L., Miller C.S. (2010). Rheumatoid arthritis and salivary biomarkers of periodontal disease. J. Clin. Periodontol..

[48] Ganzetti G., Campanati A., Santarelli A., Pozzi V., Molinelli E., Minnetti I., Brisigotti V., Procaccini M., Emanuelli M., Offidani A. (2015). Involvement of the oral cavity in psoriasis: Results of a clinical study. Br. J. Dermatol..

[49] Lehmann A.P., Nijakowski K., Swora-Cwynar E., Łuczak J., Czepulis N., Surdacka A. (2020). Characteristics of salivary inflammation in obesity. Pol. Arch. Intern. Med..

[50] Drążewski D., Grzymisławska M., Korybalska K., Czepulis N., Grzymisławski M., Witowski J., Surdacka A. (2017). Oral health status of patients with lysosomal storage diseases in Poland. Int. J. Environ. Res. Public Health.

[51] Surdacka A., Ciężka E., Pioruńska-Stolzmann M., Wender-Ożegowska E., Korybalska K., Kawka E., Kaczmarek E., Witowski J. (2011). Relation of salivary antioxidant status and cytokine levels to clinical parameters of oral health in pregnant women with diabetes. Arch. Oral Biol..

[52] Grottrup-Wolfers E., Moeller J., Karbach U., Muller-Lissner S., Endres S. (1996). Elevated cell-associated levels of interleukin 1beta and interleukin 6 in inflamed mucosa of inflammatory bowel disease. Eur. J. Clin. Invest..

[53] Reinecker H.C., Steffen M., Witthoeft T., Pflueger I., Schreiber S., MacDermott R.P., Raedler A. (1993). Enhanced secretion of tumour necrosis factor-alpha, IL-6, and IL-1 beta by isolated lamina propria mononuclear cells from patients with ulcerative colitis and Crohn’s disease. Clin. Exp. Immunol..

[54] Hirano T. (1998). Interleukin 6 and its receptor: Ten years later. Int. Rev. Immunol..

[55] Gorelik L., Flavell R.A. (2002). Transforming growth factor-beta in T-cell biology. Nat. Rev. Immunol..

[56] Lúdvíksson B.R., Gunnlaugsdóttir B. (2003). Transforming growth factor-beta as a regulator of site-specific T-cell inflammatory response. Scand. J. Immunol..

[57] Bartolomé R.A., Sanz-Rodríguez F., Robledo M.M., Hidalgo A., Teixidó J. (2003). Rapid up-regulation of alpha4 integrin-mediated leukocyte adhesion by transforming growth factor-beta1. Mol. Biol. Cell.

[58] van Harten R.M., van Woudenbergh E., van Dijk A., Haagsman H.P. (2018). Cathelicidins: Immunomodulatory antimicrobials. Vaccines (Basel).

[59] Wiesner J., Vilcinskas A. (2010). Antimicrobial peptides: The ancient arm of the human immune system. Virulence.

[60] Ellison R.T., Giehl T.J. (1991). Killing of gram-negative bacteria by lactoferrin and lysozyme. J. Clin. Investig..

[61] Abraham B.P., Thirumurthi S. (2009). Clinical significance of inflammatory markers. Curr. Gastroenterol. Rep..

[62] Lewis B.P., Burge C.B., Bartel D.P. (2005). Conserved seed pairing, often flanked by adenosines, indicates that thousands of human genes are microRNA targets. Cell.

[63] Papagiannakopoulos T., Kosik K.S. (2008). MicroRNAs: Regulators of oncogenesis and stemness. BMC Med..

[64] Liang Y., Ridzon D., Wong L., Chen C. (2007). Characterization of microRNA expression profiles in normal human tissues. BMC Genom..

[65] Pauley K.M., Satoh M., Chan A.L., Bubb M.R., Reeves W.H., Chan E.K. (2008). Upregulated miR-146a expression in peripheral blood mononuclear cells from rheumatoid arthritis patients. Arthritis Res. Ther..

[66] Dai Y., Huang Y.-S., Tang M., Lv T.-Y., Hu C.-X., Tan Y.-H., Xu Z.-M., Yin Y.-B. (2007). Microarray analysis of microRNA expression in peripheral blood cells of systemic lupus erythematosus patients. Lupus.

[67] Mitchell P.S., Parkin R.K., Kroh E.M., Fritz B.R., Wyman S.K., Pogosova-Agadjanyan E.L., Peterson A., Noteboom J., O’Briant K.C., Allen A. (2008). Circulating microRNAs as stable blood-based markers for cancer detection. Proc. Natl. Acad. Sci. USA.

[68] Wu F., Zhang S., Dassopoulos T., Harris M.L., Bayless T.M., Meltzer S.J., Brant S.R., Kwon J.H. (2010). Identification of microRNAs associated with ileal and colonic Crohn’s disease. Inflamm. Bowel Dis..

[69] Wu F., Guo N.J., Tian H., Marohn M., Gearhart S., Bayless T.M., Brant S.R., Kwon J.H. (2011). Peripheral blood microRNAs distinguish active ulcerative colitis and Crohn’s disease. Inflamm. Bowel Dis..

[70] Coskun M., Bjerrum J.T., Seidelin J.B., Nielsen O.H. (2012). MicroRNAs in inflammatory bowel disease--pathogenesis, diagnostics and therapeutics. World J. Gastroenterol..

[71] Duttagupta R., DiRienzo S., Jiang R., Bowers J., Gollub J., Kao J., Kearney K., Rudolph D., Dawany N.B., Showe M.K. (2012). Genome-wide maps of circulating miRNA biomarkers for ulcerative colitis. PLoS ONE.

[72] Lin J., Welker N.C., Zhao Z., Li Y., Zhang J., Reuss S.A., Zhang X., Lee H., Liu Y., Bronner M.P. (2014). Novel specific microRNA biomarkers in idiopathic inflammatory bowel disease unrelated to disease activity. Mod. Pathol..

[73] Namwat N., Chusorn P., Loilome W., Techasen A., Puetkasichonpasutha J., Pairojkul C., Khuntikeo N., Yongvanit P. (2012). Expression profiles of oncomir miR-21 and tumor suppressor let-7a in the progression of opisthorchiasis-associated cholangiocarcinoma. Asian Pac. J. Cancer Prev..

[74] Folini M., Gandellini P., Longoni N., Profumo V., Callari M., Pennati M., Colecchia M., Supino R., Veneroni S., Salvioni R. (2010). miR-21: An oncomir on strike in prostate cancer. Mol. Cancer.

[75] Humeau M., Vignolle-Vidoni A., Sicard F., Martins F., Bournet B., Buscail L., Torrisani J., Cordelier P. (2015). Salivary microRNA in pancreatic cancer patients. PLoS ONE.

[76] Liu X., Sempere L.F., Ouyang H., Memoli V.A., Andrew A.S., Luo Y., Demidenko E., Korc M., Shi W., Preis M. (2010). MicroRNA-31 functions as an oncogenic microRNA in mouse and human lung cancer cells by repressing specific tumor suppressors. J. Clin. Investig..

[77] Lv C., Li F., Li X., Tian Y., Zhang Y., Sheng X., Song Y., Meng Q., Yuan S., Luan L. (2017). MiR-31 promotes mammary stem cell expansion and breast tumorigenesis by suppressing Wnt signaling antagonists. Nat. Commun..

[78] Yu D., Tan A.H.-M., Hu X., Athanasopoulos V., Simpson N., Silva D.G., Hutloff A., Giles K.M., Leedman P.J., Lam K.P. (2007). Roquin represses autoimmunity by limiting inducible T-cell co-stimulator messenger RNA. Nature.

[79] Cui T.X., Kryczek I., Zhao L., Zhao E., Kuick R., Roh M.H., Vatan L., Szeliga W., Mao Y., Thomas D.G. (2013). Myeloid-derived suppressor cells enhance stemness of cancer cells by inducing microRNA101 and suppressing the corepressor CtBP2. Immunity.

[80] Vlassov A.V., Magdaleno S., Setterquist R., Conrad R. (2012). Exosomes: Current knowledge of their composition, biological functions, and diagnostic and therapeutic potentials. Biochim. Biophys. Acta.

[81] Bach D.-H., Hong J.-Y., Park H.J., Lee S.K. (2017). The role of exosomes and miRNAs in drug-resistance of cancer cells. Int. J. Cancer.

[82] Simpson R.J., Lim J.W., Moritz R.L., Mathivanan S. (2009). Exosomes: Proteomic insights and diagnostic potential. Expert Rev. Proteomics.

[83] Ritchie A.J., Crawford D.M., Ferguson D.J.P., Burthem J., Roberts D.J. (2013). Normal prion protein is expressed on exosomes isolated from human plasma. Br. J. Haematol..

[84] Zheng X., Chen F., Zhang J., Zhang Q., Lin J. (2014). Exosome analysis: A promising biomarker system with special attention to saliva. J. Membr. Biol..

[85] Hu X., Chen W., Wang D., Shi Q.-L., Zhang F.-B., Liao Y.-Q., Jin M., He C. (2008). The proteasome subunit PSMA7 located on the 20q13 amplicon is overexpressed and associated with liver metastasis in colorectal cancer. Oncol. Rep..

[86] Minelli A., Magri C., Barbon A., Bonvicini C., Segala M., Congiu C., Bignotti S., Milanesi E., Trabucchi L., Cattane N. (2015). Proteasome system dysregulation and treatment resistance mechanisms in major depressive disorder. Transl. Psychiatry.

[87] Mandel A.L., des Gachons C.P., Plank K.L., Alarcon S., Breslin P.A.S. (2010). Individual differences in AMY1 gene copy number, salivary α-amylase levels, and the perception of oral starch. PLoS ONE.

[88] Perry G.H., Dominy N.J., Claw K.G., Lee A.S., Fiegler H., Redon R., Werner J., Villanea F.A., Mountain J.L., Misra R. (2007). Diet and the evolution of human amylase gene copy number variation. Nat. Genet..

[89] Castle D., Castle A. (1998). Intracellular transport and secretion of salivary proteins. Crit. Rev. Oral Biol. Med..

[90] Ishikawa Y., Cho G., Yuan Z., Skowronski M.T., Pan Y., Ishida H. (2006). Water channels and zymogen granules in salivary glands. J. Pharmacol. Sci..

[91] Proctor G.B., Carpenter G.H. (2007). Regulation of salivary gland function by autonomic nerves. Auton. Neurosci..

[92] Nater U.M., Rohleder N. (2009). Salivary alpha-amylase as a non-invasive biomarker for the sympathetic nervous system: Current state of research. Psychoneuroendocrinology.

[93] Boissé L., Chisholm S.P., Lukewich M.K., Lomax A.E. (2009). Clinical and experimental evidence of sympathetic neural dysfunction during inflammatory bowel disease. Clin. Exp. Pharmacol. Physiol..

[94] Pellissier S., Dantzer C., Mondillon L., Trocme C., Gauchez A.-S., Ducros V., Mathieu N., Toussaint B., Fournier A., Canini F. (2014). Relationship between vagal tone, cortisol, TNF-alpha, epinephrine and negative affects in Crohn’s disease and irritable bowel syndrome. PLoS ONE.

[95] Straub R.H., Wiest R., Strauch U.G., Härle P., Schölmerich J. (2006). The role of the sympathetic nervous system in intestinal inflammation. Gut.

[96] Voganatsi A., Panyutich A., Miyasaki K.T., Murthy R.K. (2001). Mechanism of extracellular release of human neutrophil calprotectin complex. J. Leukoc. Biol..

[97] Ryckman C., Vandal K., Rouleau P., Talbot M., Tessier P.A. (2003). Proinflammatory activities of S100: Proteins S100A8, S100A9, and S100A8/A9 induce neutrophil chemotaxis and adhesion. J. Immunol..

[98] Sopalla C., Leukert N., Sorg C., Kerkhoff C. (2002). Evidence for the involvement of the unique C-tail of S100A9 in the binding of arachidonic acid to the heterocomplex S100A8/A9. Biol. Chem..

[99] Turovskaya O., Foell D., Sinha P., Vogl T., Newlin R., Nayak J., Nguyen M., Olsson A., Nawroth P.P., Bierhaus A. (2008). RAGE, carboxylated glycans and S100A8/A9 play essential roles in colitis-associated carcinogenesis. Carcinogenesis.

[100] Vogl T., Tenbrock K., Ludwig S., Leukert N., Ehrhardt C., van Zoelen M.A.D., Nacken W., Foell D., van der Poll T., Sorg C. (2007). Mrp8 and Mrp14 are endogenous activators of Toll-like receptor 4, promoting lethal, endotoxin-induced shock. Nat. Med..

[101] D’Haens G., Ferrante M., Vermeire S., Baert F., Noman M., Moortgat L., Geens P., Iwens D., Aerden I., Van Assche G. (2012). Fecal calprotectin is a surrogate marker for endoscopic lesions in inflammatory bowel disease. Inflamm. Bowel Dis..

[102] Tibble J.A., Sigthorsson G., Bridger S., Fagerhol M.K., Bjarnason I. (2000). Surrogate markers of intestinal inflammation are predictive of relapse in patients with inflammatory bowel disease. Gastroenterology.

[103] Cuida M., Halse A.K., Johannessen A.C., Tynning T., Jonsson R. (1997). Indicators of salivary gland inflammation in primary Sjogren’s syndrome. Eur. J. Oral Sci..

[104] Haririan H., Andrukhov O., Pablik E., Neuhofer M., Moritz A., Rausch-Fan X. (2016). Comparative analysis of Calcium-Binding Myeloid-Related Protein-8/14 in saliva and serum of patients with periodontitis and healthy individuals. J. Periodontol..

[105] Moher D., Shamseer L., Clarke M., Ghersi D., Liberati A., Petticrew M., Shekelle P., Stewart L.A. (2015). PRISMA-P Group Preferred reporting items for systematic review and meta-analysis protocols (PRISMA-P) 2015 statement. Syst. Rev..

[106] Study Quality Assessment Tools | NHLBI, NIH. https://www.nhlbi.nih.gov/health-topics/study-quality-assessment-tools.

[107] OCEBM Levels of Evidence. https://www.cebm.net/2016/05/ocebm-levels-of-evidence/.

